# CT-Screening for lung cancer does not increase the use of anxiolytic or antidepressant medication

**DOI:** 10.1186/1471-2407-12-188

**Published:** 2012-05-23

**Authors:** Linda Kaerlev, Maria Iachina, Jesper Holst Pedersen, Anders Green, Bente Mertz Nørgård

**Affiliations:** 1 Research Unit of Clinical Epidemiology, Institute of Clinical Research, University of Southern Denmark, Odense, Denmark; 2 Centre for National Clinical Databases South, Odense University Hospital, Odense, Denmark; 3 Department of Cardiothoracic Surgery RT, Rigshospitalet, University of Copenhagen, Copenhagen, Denmark

**Keywords:** Lung cancer, Screening, Randomised trial, Psychosocial distress

## Abstract

**Background:**

CT screening for lung cancer has recently been shown to reduce lung cancer mortality, but screening may have adverse mental health effects. We calculated risk ratios for prescription of anti-depressive (AD) or anxiolytic (AX) medication redeemed at Danish pharmacies for participants in The Danish Lung Cancer Screening Trial (DLCST).

**Methods:**

The DLCST was a randomized clinical trial which comprised 4,104 former or present smokers who were randomized from 12 May 2004 to 20 June 2006 to either CT scan of the chest, lung-function test and filling in questionnaires annually for five years in the period 1 April 2006–31 March 2010 (n = 2,052), or to a control group (n = 2,052) receiving similar procedures except CT scan. We used CT scan intervention group versus control group status as exposure. The follow-up period for use of AD or AX was three years. Baseline data on civil status, socioeconomic status, and co-morbidity as well as outcome data on AD and AX were obtained by linkage to national registries.

**Results:**

The intervention and the control groups did not differ by age, gender, civil status, socio-economic position, co-morbidity index or former use of AD or AX. The adjusted risk ratio for at least one recipe of AD or AX in the CT intervention group during follow-up was not increased when adjusting for previous use of AD or AX, HR 1.00, 95 % CI (0.90-1.12). Similar results were seen when excluding subjects using AD or AX in a four-month or two-year period before baseline, when analyzing AD and AX separately, or requiring at least two recipes.

**Conclusions:**

We found no indications that participation in a lung cancer CT-screening program increases the risk of specific adverse mental health outcomes.

**Trial registration:**

Clinical Trials.gov Protocol Registration System (NCT00496977).

## Background

Screening for serious diseases in occupational or lifestyle related target groups has been more common in the recent years. The recent demonstration of a reduction in lung cancer specific mortality by more than 20 per cent and an overall 7 % reduced mortality following CT screening for lung cancer among tobacco smokers may have great implications for health practice. [1] Before a general implementation of CT screening, it will be necessary also to clarify any adverse effects of CT screening. While experiencing a cancer diagnosis obviously may cause anxiety or even depression, the risk of developing depressive or anxiety symptoms following cancer screening has only been investigated in a few studies. [2-4] Participation in screening might be related to gender, age, co-morbidity, and social factors that are related both to tobacco smoking as well as later mental health. [5-8] Therefore, the possibility of biased risk estimates of developing depressive or anxiety symptoms might occur unless such potential confounding factors are taken into account. Furthermore, five out of six previous studies of participation bias, among volunteers in medical trials have shown that participants in screening programs, in general, have a more robust personality and less psychopathology compared with smokers in the general population. [6,9-13] This study aimed to examine psychological adverse effects in a Danish randomized clinical trial (The Danish Lung Cancer Screening Trial (DLCST)) with the calculation of risk ratios for prescription of anti-depressive (AD) or anxiolytic (AX) medication according to CT screening intervention or control group status with adjusting for potential confounding factors at baseline.

## Methods

### The DLCST

The study is based on the DLCST - a randomized clinical trial - with the overall aim to assess whether CT-screening of heavy tobacco smokers or ex-smokers with a history of at least 20 pack-years can reduce mortality from lung cancer. A total of 4,104 volunteers aged between 50 and 70 years, were included in the DLCST, with randomization at baseline - a specific date during the period 12 May 2004 until 20 June 2006- to either an intervention group (2,052 persons) or a control group (2,052 persons) followed by four annual screening rounds in the period 1 April 2006–31 March 2010. [5].

### A baseline CT scan was performed in all participants of the CT intervention group

Pulmonary nodules were classified according to size, morphology and growth, and the assessment was validated by two radiologists. [5] At base line (1) nodules smaller than 5 mm and calcified (benign) nodules were tabulated, (2) non-calcified nodules between 5 and 15 mm were rescanned after three months. If the nodule increased in size or was larger than 15 mm the participant was referred for diagnostic procedures. The growth of nodules was assessed by both linear measurement and volumetric analysis. [14] On an annual basis the CT intervention group was offered a CT scan and completed annual lung function tests together with questionnaires on smoking habits, health status, psychosocial factors, and quality of life issues. The control group had identical procedures as the screening group, but without CT scans.

Persons with a permanent address in Denmark have a unique 10-digit Civil Registration Number (CPR), which has been assigned to each Danish resident since 1968. The number includes information on birthday and sex, and it is used by all authorities for registration purposes. We used the CPR to link the DLCST project data with public registries including The Danish Civil Registration System [15], The Danish National Patient Register [16], and with public registries developed by Statistics Denmark such as The Household and Family Statistics (based on The Danish Civil Registration System) as well as The personal income statistics [17] and The Danish National Prescription Registry of the Danish Medicines Agency. [18].

Both the intervention group and the control group in the DLCST were followed-up for three years for use of AD or AX, counted from the baseline date of randomization in the DLCST.

### Demographic characteristics and co-morbidity of participants

The Danish Civil Registration System contains information on gender, addresses, dates of birth, and death and migration for every person who is or has been a Danish resident at any time between 1968 and the present. Information on civil status and socio-economic position at randomization (study baseline) was obtained from public registries in Statistics Denmark. Civil status was defined as living alone versus all others. Socio-economic position (SES) was defined by level of yearly income and included in the analyses as a categorical variable in three categories (below DKK 250,000, DKK 250–350,000, and above DKK 350,000). Information on co-morbidity was obtained from the NPR with calculation of a slight modification of the Charlson Co-morbidity Index (CCI). [19,20].

The CCI was re-coded into a categorical variable (0, 1, and above 1 point on the CCI scale). Smoking status at baseline as a categorical variable (current or former smoker) was used as a confounder in supplementary analyses.

### Measures of outcome

AD and AX are only available by prescription in Denmark. Using the unique PIN, we obtained complete information about redeemed AD and AX prescriptions by linkage to The Danish National Prescription Registry. [18] This register covers all pharmacies in Denmark and classifies prescribed pharmaceuticals according to the Anatomical Therapeutic Chemical classification system (ATC) at the level of the generic pharmaceutical. We used prescription of one or more of the following drugs to define the endpoints for the present study: The AD group comprised the overall ATC-group “N06A”, including tricyclic antidepressants (TCA, ATC code N06AA), selective serotonin reuptake inhibitors (SSRI, ATC code N06AB), noradrenalin reuptake inhibitors (NARI, ATC code N06AX) and monoamine oxidase inhibitors (MAO-inhibitors, ATC codes N06AF and N06AG). [21].

Lithium salts are mostly prescribed for bipolar affective disorders and were not included. Zyban has both an anti-depressive effect (ATC code N 06 AX 12) and is used as a smoking-cessation drug. However, none of the DLCST participants received prescribed Zyban in the follow up period, and therefore Zyban was not included in the outcome. The AX group comprised the overall ATC group “NO5B”: Benzodiazepine derivatives (ATC code N05BA), Diphenylmethane derivatives (ATC code N05BB), Carbamates (ATC code N05BC), Dibenzo-bicyclo-octadiene derivatives (ATC code N05BD), Azaspirodecanedione derivatives (ATC code N05BE), and other anxiolytic medications (ATC code N05BX).

According to guidelines from the Danish health authorities the first-line drug for anxiety disorders in Denmark is SSRI antidepressants. Using prescription of AD or AX as a proxy for medical conditions, without information of the underlying medical condition on the recipes to clearly separate depressive disorders from anxiety disorders, did not allow us to conclude on separate analyses of AD and AX. We have thus presented the results for the analysis of AD or AX as our main results.

### Analysis

First, we compared the screening intervention group with the controls at baseline with respect to demographic characteristics with chi-squared tests, except for continuous variables, which were compared between the groups with Students *t*-test. Secondly, we analyzed the risk ratio for prescription of AD or AX medication, redeemed at pharmacies at least once during a three year follow-up period, from baseline by proportional hazard regression with adjustment for use of AD and AX in the previous four months before baseline. Thirdly, we analyzed the risk ratio for prescription of AD or AX medication at least once during a three year follow-up period from baseline with exclusion at baseline of users of AD and AX in the previous four months before baseline. In addition, we performed extra analyses by extending the previous use of AD or AX to a two year period, by either analyzing AD and AX separately, or requiring at least two recipes.

The follow-up ended at the date of the prescription of the medication under study, the date of death, date of emigration, date of disappearance, date of a diagnosis of lung cancer, or after 3 years of follow-up for each individual, whichever came first. The Person-time at risk was calculated for each individual. The time during follow-up was counted in days.

Hazard ratios (HR) with 95 % confidence intervals (CIs) were calculated with adjustment for gender, age, civil status, SES, and CCI at baseline, and previous prescription of AD or AX.

In a sub-analysis, and due to the limited statistical power, we used logistic regression to test whether the persons diagnosed with lung cancer in the intervention group more often had used AD or AX compared with persons diagnosed with lung cancer in the control group.

### Ethical aspects

The Danish Data Protection Agency and the Danish Medicines Agency approved the present study (J.nr. 2008-41-2764). The DLCST was approved by the Ethical Committee of Copenhagen County on January 31, 2003 and funded in full by the Danish Ministry of Interior and Health on June 23, 2004. Approval of data management in the trial was obtained from the Danish Data Protection Agency on February 11, 2005. The trial is registered in Clinical Trials.gov Protocol Registration System (identification no. NCT00496977).

All participants gave written informed consent to participation in the Danish Lung Cancer Screening Trial, which was approved by the Ethical Committee of Copenhagen County on January 31, 2003.

## Results

A total of 4,104 persons were randomized into the intervention (2,052) or control group (2,052). Characteristics of the cohort are given in Table 1.

**Table 1:** Descriptive characteristics of participants of the DLCST at baseline in 2004

**Characteristics**	**Respondents**	**Intervention**	**Control**	**p-value**
	**N = 4,104**	**N = 2,052**	**N = 2,052**	
Gender, women, number, (%)	4,104	905 (44.10)	932 (45.42)	0.397
Mean age, years	4,104	57.35	57.31	0.807
Age group (above mean: 57.3 years) (%)	4,104	1,001 (48.78)	972 (47.37)	0.365
Civic status, living alone, number, (%)	4,076	586 (28.58)	587 (28.60)	0.980
Mean income per year (DKK)	4,100	316,910	315,814	0.859
SES group, number, (%)	4,100			
Below 250,000 DKK/year		754 (36.74)	759 (36.99)	
250–350,000 DKK/year		650 (31.68)	643 (31.34)	
Above 350,000 DKK/year		648 (31.58)	650 (31.68)	0.972
Charlson co-morbidity index at baseline, number, (%)	4,104			
			
0		1,717 (83.67)	1,693 (82.50)	0.336
1		235 (11.45)	238 (11.60)	
>1		100 (4.87)	121 (5.90)	
Prescription of AD or AX medication at least once during the 4 months period before baseline, number, (%)	4,104	269 (13.11)	277 (13.50)	0.713
Prescription of AD or AX medication at least once during the 2 years period before baseline, number, (%)	4,104	483 (23,54)	502 (24,46)	0.487

Compared to the controls, the screened intervention group did not statistically differ significantly at baseline at the time of randomization with respect to gender, mean age, age group above 57.3 years of age, civil status with percentage living alone, SES mean income, SES - percentage within the lowest, the middle, or within the highest income group, percentage with CCI above 1 at the time of randomization, or with respect to percentage having either AD or AX medication prescribed at least once during the four months period before baseline, or at least once during the two years period before baseline (Table 1).

Observations were censored if the participant died (n = 121; 67 in the intervention group, 54 controls), disappeared (1 in the intervention group), or emigrated (n = 28; 14 in the intervention group, 14 controls) during follow-up, or were diagnosed with lung cancer (n = 92; 68 in the intervention group, 24 controls). The mean follow-up time with standard deviation (SD) was 854 (SD 404) days for the intervention group and 851 days (SD 409) for controls, and the median follow-up time was 1096 days (3 years) for both groups.

Altogether 30.90 % (n = 634) of the intervention group and 31.38 % (n = 644) of the controls have had at least one redeemed prescription of AD or AX during follow up (former users not excluded), Pearson chi2 = 0.1136, Pr = 0.736.

### The unadjusted risk estimates for AD or AX in the intervention group and in the control group are shown in Figure 1

**Figure 1:**
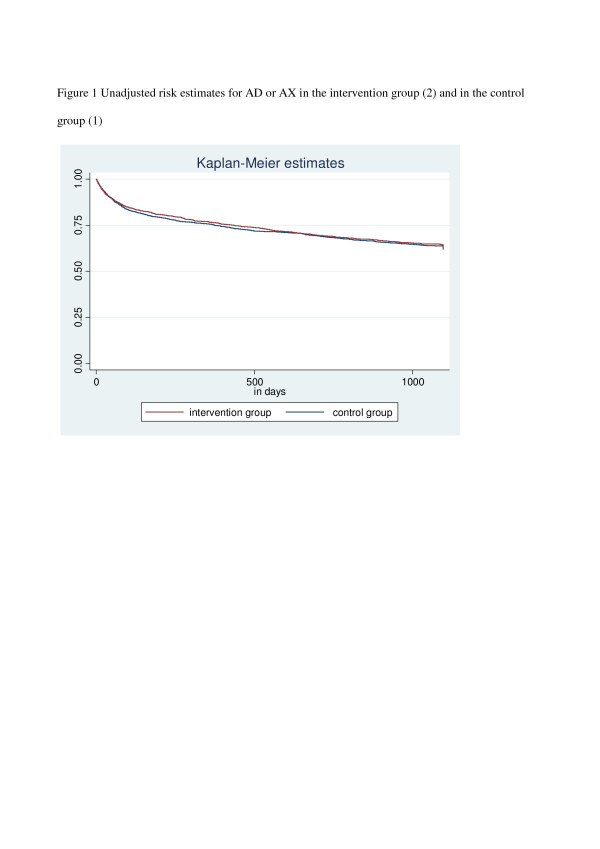
Unadjusted risk estimates for AD or AX in the intervention group (2) and in the control group (1).

The risk of at least one redeemed prescription of AD or AX among CT scan screened subjects during follow-up according to age group, gender, civil status, SES group, and CCI group, and without exclusion at baseline of former users of AD and AX in the previous four months before baseline is provided in Table 2. No differences between the two groups were seen, neither when adjusting for previous use of AD or AX, (HR 1.00, 95 % CI 0.90-1.12), nor when excluding subjects using AD or AX in a four months period before baseline HR 1.01, 95 % CI (0.88-1.17) (Table 3) or excluding a two year period before baseline, when analyzing AD and AX separately, or requiring at least two recipes (data not shown).

**Table 2:** Hazard ratio (HR) for use of antidepressants or anxiolytic drugs in the intervention group compared with controls adjusted for former use of AD or AX, and other confounders

	**Relative risk estimates**			
	**N, (%)**	**Adj. HR**	**95 % CI**	
Intervention versus control status				
Control	644 (31.38)	1.00	Reference	
Intervention	634 (30.90)	1.00	0.90	1.12
Possible confounders:				
Gender				
Men	561 (43.90)	1.00	Reference	
Women	717 (56.10)	1.36	1.21	1.53
Age				
≤ mean age 57.3 years	685 (32.14)	1.00	Reference	
>mean age 57.3 years	593 (30.06)	0.92	0.82	1.03
Civil status				
Living together	806 (63.46)	1.00	Reference	
Living alone	464 (36.54)	1.26	1.12	1.42
Social status (SES)				
Low	576 (38.07)	1.00	Reference	
Middle	397 (30.70)	0.86	0.80	0.93
High	305 (23.50)	0.43	0.37	0.49
Charlson Co-morbidity index (CCI)				
0	985 (28.89)	1.00	Reference	
1	197 (41.65)	1.25	1.14	1.37
> 1	96 (43.44)	2.50	2.38	2.62
Prescription of AD or AX at least once during the 4 months period before baseline				
No	775 (21.78)	1.00	Reference	
Yes	503 (92.12)	18.52	16.12	21.27
Total 1,278 (31.14)				

**Table 3:** Hazard ratio (HR) for use of antidepressants or anxiolytic drugs in the intervention group compared with controls, with exclusion at baseline of former users of AD or AX, and adjusted for confounders

	**Relative risk estimates**			
	**N, (%)**	**Adj. HR**	**95 % CI**	
Intervention versus control status				
Control	385 (21.69)	1.00	Reference	
Intervention	390 (21.87)	1.01	0.88	1.17
Possible confounders:				
Gender				
Men	367(47.35)	1.00	Reference	
Women	408(52.65)	1.69	1.45	1.96
Age				
≤ mean age 57.3 years	415 (53.55)	1.00	Reference	
>mean age 57.3 years	360 (46.45)	0.88	0.76	1.03
Civil status				
Living together	518 (67.19)	1.00	Reference	
Living alone	253 (32.81)	1.30	1.11	1.52
Social status (SES)				
Low	313 (40.39)	1.00	Reference	
Middle	253 (32.65)	0.84	0.77	0.92
High	209 (26.97)	0.42	0.34	0.50
Charlson Co-morbidity index (CCI)				
0	609 (78.58)	1.00	Reference	
1	111 (14.32)	1.35	1.20	1.52
> 1	55 (7.10)	2.70	1.13	4.27
Total 775 (21.78)				

Supplementary analyses with further adjustment for smoking status at baseline (current or former smoker) were performed, and no significant differences in the HR for AD or AX were seen before and after adjustment. Similarly, the HRs for the covariates only changed slightly in Table 2 and 3 after further adjustment for smoking status at baseline.

Among the 92 persons with a lung cancer diagnosis (68 in the CT scan intervention group; 24 in the control group), 52.4 % of the control group and 37.1 % of the intervention group had used AD or AX. Among the persons without a cancer diagnosis 31.2 % of the control group and 30.7 % of the intervention group had used AD and AX, odds ratio 0.48; 95 % CI (0.17-1.37). Thus, no statistically significant difference was seen for use of AD or AX between lung cancer patients in the CT scan intervention group and the control group, and with a very broad confidence interval due to the limited number of cases. Further adjustment for former use of AD or AX at baseline did not change this finding. We did not make an additional sub-analysis with exclusion at baseline of former users of AD or AX, since the number of lung cancer cases in the intervention group (n = 55) and in the control group (n = 19) after this exclusion was small. All the sub-analyses on persons diagnosed with lung cancer had too few cases for proportional hazard regression analyses with adjustment for the full model of confounders.

## Discussion

We found no indications that participation in a lung cancer CT-screening program increases the risk of specific adverse mental health outcomes, measured by prescription of AD or AX as a proxy for medical conditions. At baseline,- an individual day in the recruitment period,- the screened study subjects did not differ from controls by gender, age group, civil status, SES group, CCI group, or previous use of AD or AX, and thus, the randomization was successful. During follow-up no differences in the use of AD or AX between the two groups were found. Our findings are in line with a similar study investigating possible adverse effects of participating in screening programs, but they need cautious interpretation. [2] Although we found no indication of an increased use of AD or AX in the intervention group compared to the control group, our findings for the present outcomes do not exclude an association for other adverse effects. Bunge et al. found that participants with a high affective risk perception showed higher lung cancer-specific distress than participants with a low affective risk perception, both at baseline and six months after screening, and therefore attention for this specific group was recommended. [4] The strengths of our study were that comprehensive measures of potential confounders were available for the entire cohort. Furthermore, complete and independent information on the chosen outcome prescription of AD or AX and possible confounders such as demographic factors and CCI were available for the entire study population from the Danish registries.

A limitation of the present study was that we used prescriptions of AD or AX as a proxy for medical conditions without information of the underlying medical condition. Since we expect, that only more severe degrees of depression and anxiety disorders were medically treated, our study is not expected to include a milder degree of depression and anxiety disorders. Furthermore, the lack of information of the underlying medical condition on the recipes to clearly separate depressive disorders from anxiety disorders, did not allow us to conclude on separate analyses of AD and AX. In addition, it was a limitation that we were not able to stratify for or adjust for personal characteristics (robustness in personality and psychopathology) at baseline, but we believe that randomization have reduced selection bias due to this factor.

Whether the DLCST screening population is comparable to the general Danish population of smokers or ex-smokers, has been studied by Hestbech et al. by comparison with a matched population sample. [6] Participants in DLCST were found to have a higher socio-economic status and less negative psychosocial aspects than the population sample, together with differences regarding age, gender and geographical area. Therefore the participants of DLCST may be more robust than the general population. The National Lung Screening Trial (NLST) from the United States, which showed a large reduction in mortality among screened participants, also showed that participants were better educated than a comparable sample from the general population. [1] In the NELSON trial no participation bias was found. [22,23] However, selection bias due to selected recruitment at baseline has been shown, in a recent publication, not to play a major role for the risk estimates between psychosocial factors and AD and AX medication during follow up. [24] Furthermore, we found that 30.90 % of the cases and 31.38 % of the controls in the DLCST had used AD or AX either before or during the follow-up period. This does not point to a very robust participation group, but needs to be studied in comparison with the prescription pattern in an external reference group.

## Conclusions

In conclusion, despite the use of several analytic strategies, we found no indications that participation in a Danish lung cancer CT-screening program lead to changes in prescription of antidepressants and anxiolytic medications. This indicates that CT screening in general does not cause major psychological disturbances. However it may have other psycho-social consequences not reflected in the pattern of medications.

## Competing interests

The authors declare that they have no competing interests.

## Authors’ contributions

LK drafted the manuscript and MI and LK performed the statistical analysis for the present paper. JHP, BMN, and AG have helped with the interpretation of the analyses and with revising the manuscript critically. JHP is principal investigator of the DLCST. All authors participated in the design and coordination of the present study, and have made substantial contributions to the interpretation of data, and have read and approved the final manuscript.

## Pre-publication history

The pre-publication history for this paper can be accessed here:

http://www.biomedcentral.com/1471-2407/12/188/prepub
